# Primary Scientific Literature Represents an Essential Source of Telomeric Repeat Sequences

**DOI:** 10.1128/spectrum.01230-23

**Published:** 2023-06-13

**Authors:** Filip Červenák, Regina Sepšiová, Vratislav Peška, Jozef Nosek, Ľubomír Tomáška

**Affiliations:** a Department of Genetics, Faculty of Natural Sciences, Comenius University in Bratislava, Bratislava, Slovakia; b Department of Cell Biology and Radiobiology, Institute of Biophysics of the Czech Academy of Sciences, Brno, Czech Republic; c Department of Biochemistry, Faculty of Natural Sciences, Comenius University in Bratislava, Bratislava, Slovakia; The Ohio State University

**Keywords:** evolution, fungi, telomere

## LETTER

Sun et al. (1) report on development of TelFinder, a Python script for identification of telomeric repeats from genomic data. Although we appreciate the potential utility of the tool, we identified several inconsistencies between the claims of the authors and available published results. (i) The NCBI genome database lists genomic data for more than 4,000 fungal species (https://www.ncbi.nlm.nih.gov/genome/), i.e., substantially more than reported by Sun et al. (ii) There are several erroneous taxonomical assessments. For example, Saccharomycopsis malanga and Saccharomycopsis fibuligera belong not to the genus *Scheffersomyces* (as shown in Fig. 3) but to *Saccharomycopsis* (2). Similarly, Yarrowia lipolytica is not a member of the genus *Metschnikowia* (family *Metschnikowiaceae*) but, along with other species of the genus *Yarrowia*, is classified in a separate family, *Dipodascaceae* (3). (iii) Some “identified” telomeric repeats do not correspond to those that were already known and experimentally validated. For example, Candida albicans telomeres were characterized 30 years ago (4) and consist of an array of 5′-TGTACGGATGTCTAACTTCTTGG-3′ repeats (not a sequence depicted in Fig. 2). Furthermore, in contrast to claims of Sun et al., the C. albicans telomeric motif is different from that of C. tropicalis (5′-GTGTAAGGATGTCACGATCATTG-3′), whose strains may carry two variants (5′-GTGTA[C/A]GGATGTCACGATCATTG-3′ [5]). Similarly, Kluyveromyces lactis (5′-TTTGATTAGGTATGTGGTGTACGGA-3′) and K. marxianus (5′-TTTGATTAGTTATGTGGTGTACGGA-3′) do not share the same telomeric motif (5). Strangely, Table S1 lists the telomeric motifs identified in these studies as being “in accordance” with those reported by the authors. (iv) The authors compare their results with the Telomerase Database (http://telomerase.asu.edu), which is far from being complete. For example, the sequence of telomeric repeat of Y. lipolytica has been known since 2010 (6), yet the database still contains an incorrect version. Furthermore, a study published nearly 15 years ago identified telomeric repeats in a number of *Candida* spp. (7), yet telomeric sequences of only two *Candida* spp. are included in the database. In this context, the statement of Sun et al. that their “finding suggests that the telomeric repeats of fungi can vary within one genus” is certainly true, but this has been known for a long time. The second problem with referencing the Telomerase Database is that it is outdated, and there are recent studies substantially expanding the list of known telomeric repeats. For example, two papers (8, 9) identified candidate telomeric motifs in more than 200 fungal species (specifically those belonging to Saccharomycotina and Taphrinomycotina), many of which were “rediscovered” by Sun et al. (1) and claimed to be novel. These studies as well as earlier publications (7, 10) also report the telomeric motifs for species that are shown in Fig. 2 without the motif (e.g., C. orthopsilosis, Hyphopichia burtonii, Millerozyma farinosa, and Naumovozyma castellii).

In summary, we believe that the ignorance of primary literature and relying on a single outdated and incomplete source not only compromises the conclusions of the discussed paper but may also lead to confusion for the readers.
